# Ionizing Radiation Reduces Head and Neck Squamous Cell Carcinoma Cell Viability and Is Associated with Predictive Tumor-Specific T Cell Responses

**DOI:** 10.3390/cancers15133334

**Published:** 2023-06-25

**Authors:** Puja Upadhaya, Nathan Ryan, Peyton Roth, Travis Pero, Felipe Lamenza, Anna Springer, Pete Jordanides, Hasan Pracha, Darrion Mitchell, Steve Oghumu

**Affiliations:** 1 Department of Pathology, The Ohio State University Wexner Medical Center, Columbus, OH 43210, USA; upadhaya.1@osu.edu (P.U.); ryan.1046@osu.edu (N.R.); roth.633@buckeyemail.osu.edu (P.R.); lamenza.1@buckeyemail.osu.edu (F.L.); springer.267@buckeyemail.osu.edu (A.S.); jordanides.2@buckeyemail.osu.edu (P.J.); pracha.3@buckeyemail.osu.edu (H.P.); 2 College of Dentistry, The Ohio State University, Columbus, OH 43210, USA; pero.17@osu.edu; 3 Department of Microbiology, The Ohio State University, Columbus, OH 43210, USA; 4 Department of Radiation Oncology, The Ohio State University Wexner Medical Center, Columbus, OH 43210, USA; darrion.mitchell@osumc.edu

**Keywords:** oral cancer, HNSCC, ionizing radiation, radiation therapy

## Abstract

**Simple Summary:**

Today’s standard of treatment for advanced head and neck squamous cell carcinoma (HNSCC) is invasive surgical resection. Radiation co-therapy has received a great deal of attention for its ability to reduce tumor recurrence, common in these cancers. The aim of our study was to investigate the ability of tumor-targeted radiation therapy to generate tumor antigens capable of eliciting host immune response pathways. Using a murine model of HNSCC with tumor cells injected into immune-competent mice, we determined that antigens generated by the irradiation of tumor cells induced potent immune responses compared to other methods for tumor antigen preparations. This response was also found to display comparable sensitivity, but greater specificity for T cell activation compared to conventional anti-CD3 and CD28 antibodies. This information may be utilized to predict patient responses to radiation and immune therapy.

**Abstract:**

Head and neck squamous cell carcinoma (HNSCC) is common and deadly, and there is a need for improved strategies to predict treatment responses. Ionizing radiation (IR) has been demonstrated to improve HNSCC outcomes, but its effects on immune responses are not well characterized. We determined the impact of IR on T cell immune responses ex vivo. Human and mouse HNSCC cells were exposed to IR ranging from 20 to 200 Gy to determine cell viability and the ability to stimulate T-cell-specific responses. Lymph node cells of LY2 and MOC2 tumor-bearing or non-tumor-bearing mice were re-stimulated with a tumor antigen derived from LY2 or MOC2 cells treated with 200 Gy IR, ultraviolet (UV) exposure, or freeze/thaw cycle treatments. T cell proliferation and cytokine production were compared to T cells restimulated with plate-bound CD3 and CD28 antibodies. Human and mouse HNSCC cells showed reduced viability in response to ionizing radiation in a dose-dependent manner, and induced expression of T cell chemotactic cytokines. Tumor antigens derived from IR-treated LY2 and MOC2 cells induced greater proliferation of lymph node cells from tumor-bearing mice and induced unique T cell cytokine expression profiles. Our results demonstrate that IR induces potent tumoral immune responses, and IR-generated tumor antigens can potentially serve as an indicator of antitumor immune responses to HNSCC in ex vivo T cell restimulation assays.

## 1. Introduction

Head and neck squamous cell carcinoma (HNSCC) is a heterogeneous group of malignancies originating in the oral cavity, larynx, and pharynx. As the sixth leading cancer worldwide, it is estimated that 65,630 new cases and 14,500 deaths from HNSCC occur in the United States each year [1]. The principal risk factors for HNSCC are exposure to tobacco and alcohol and human papillomavirus (HPV) infection [2,3]. Although primary surgical resection remains the standard of care for these patients, tumor recurrence occurs in 40% of cases, and the late-stage five-year survival rate is less than 50% [4,5]. There is therefore a need for improved approaches for HNSCC management and more effective strategies to predict patient responses to treatment.

Ionizing radiation (IR) has emerged as a major treatment option to improve HNSCC outcomes. The field of radiation therapy has seen considerable recent advancement and ionizing radiation (IR) has been widely used in cancer therapy [6]. Of the several forms of IR, gamma radiation and X-ray can easily penetrate the human body [7]. Most frequently, IR therapy is supplied to the patient as high-intensity X-rays, with therapeutic doses reaching as high as 70 Gy [8]. In combination with surgical resection, patients receiving IR therapy have reported as high as 68.08% 5-year survival compared to only 31.03% survival in patients receiving resection alone [9]. However, higher doses of radiation are associated with pain at the exposure site, reduced salivation, decreased taste, difficulty swallowing, and a decrease in overall quality of life [10]. Synergizing radiotherapy with other treatment options to reduce necessary radiation exposure while delivering an enhanced or equally effective treatment is therefore a desirable outcome.

Radiation induces DNA damage, apoptosis, necrosis, and genomic instability in cancer cells [11,12]. Exposure to high-energy radiation introduces potentially lethal double-strand breaks in DNA. When cellular mechanisms are unable to repair this DNA damage, caspase-mediated apoptosis results [13,14]. Further, it has been recently demonstrated that IR is capable of inducing ferroptosis-regulated cell death in tumor cells, which is associated with an increased expression of ACSL4 [15]. IR also has an indirect cytotoxic impact on cancer cells. The generation of reactive oxygen species mediated by radiolysis of water molecules induces the generation of free hydroxyl radicals, which may in turn create double-strand breaks in tumor cell DNA initiating cellular apoptosis [16].

In addition to its cytotoxic effect on cancer cells, recent studies suggest that radiotherapy enhances the anticancer immune response. Current research has demonstrated the capacity of low-dose ionizing radiation to enhance the immunogenicity of tumors, thereby raising the possibility of synergizing radiotherapy with immunotherapy [17]. Exposure of cancer-bearing mice to IR increases CD8^+^ cytotoxic T cell recruitment and reduces immunosuppressive myeloid-derived suppressor cells (MDSCs) within the tumor microenvironment. Low-dose IR has also been demonstrated to increase the efficacy of tumor antigen processing and subsequent presentation, thereby enhancing the immunogenicity of tumors [18]. Furthermore, combined immunotherapy and radiation therapy have also been shown to generate abscopal responses systemically, reducing the incidence of cancer metastasis [19]. However, the mechanisms underlying irradiation effects on tumor immune responses within the HNSCC tumor microenvironment are not yet fully characterized. These mechanistic studies are essential in order to effectively exploit the synergistic effects of radiation and immunotherapies. Herein, we describe a model that can be used to further explore the tumor-specific immunological response to radiation therapy in HNSCC.

The potential impact of irradiation on tumor immune responses also raises the potential application of this approach to diagnostic assays of tumor antigen-specific responses. This would be potentially useful in determining the degree and specificity of tumoral immune responses in HNSCC. Using irradiated tumor cells as an antigen, T-cell-specific antitumor immune responses can be measured in cancer patients in order to determine the nature of tumor immunity as well as the potential efficacy of immunotherapy against HNSCC in the setting of radiation therapy.

The objectives of this study are to determine the impact of irradiated tumor cells on antigen-specific anti-tumor immune responses and determine the utility of irradiated HNSCC cancer cells as tumor antigens for ex vivo T-cell-based immune assays. To do this, we investigated the effect of HNSCC tumor antigens prepared by IR, ultraviolet (UV) radiation, or freeze-thawed (F/T) tumor cells, on tumor-specific T cell responses. These effects were compared to T cell responses generated by non-antigen-specific T cell receptor stimulation using CD3 and CD28 antibodies. Our results indicate that tumor cells exposed to IR are capable of inducing T cell immune responses, which, although not as robust as CD3/CD28 stimulation, can potentially serve as an indicator of antitumor immune responses in HNSCC. Our study demonstrates an optimized ex vivo model for studying the immune milieu in response to IR in the treatment of HNSCC. Characterization of the immune response to the IR-generated tumor antigen has potential applications in the emerging field of immunoradiotherapy treatment for HNSCC.

## 2. Materials and Methods

### 2.1. Cell Lines

Authenticated HPV-negative human (CAL27 and SCC83) and mouse (LY2 and MOC2) HNSCC cell lines were maintained according to specified recommendations. All media were supplemented with 2% fetal bovine serum (Corning, Corning, NY, USA), along with 100 μg/mL penicillin G, 100 μg/mL streptomycin, and 2 mM L-glutamine (Life Technologies, Carlsbad, CA, USA) at 37 °C and 5% CO_2_.

### 2.2. In Vivo Oral Cancer Cell Injections

All mice were maintained under a 12-h day/night cycle and provided access to food and water ad libitum. At six weeks of age, BALB/c mice received injections consisting of equal parts Matrigel (Corning) and 5 × 10^5^ LY2 cells in media or media without cells in the right buccal mucosa [20]. C57BL/6 mice were injected with 3 × 10^4^ MOC2 cells in 30 µL of PBS into the right buccal mucosa [21]. Animals were kept according to regulations and under the supervision of University Laboratory Animal Resources and were approved by the Institutional Animal Care and Use Committee, Protocol #2018A00000054, and Institutional Biosafety Committee of The Ohio State University.

### 2.3. Irradiation

At approximately 75% confluence, HNSCC cells were trypsinized and 2500 cells were seeded in a volume of 200 µL in triplicate in a 96-well plate. Using an RS2000 X-ray irradiator (Rad Source, Buford, GA, USA), cells were exposed to different doses of 0, 20, 50, 100, or 200 Gy of radiation. Following irradiation, cells were allowed to grow for a period of 48 h, at which time viable cells were enumerated by microscopy and image analysis performed using ImageJ.

### 2.4. Antigen Generation

LY2 and MOC2 HNSCC cells were grown in complete media and subjected to three different conditions: Exposure to 200 Gy of X-ray irradiation, 10 min of UV light exposure, and five freeze/thaw cycles alternating between dry ice and a water bath at 55 °C. Cell death was assessed using a trypan blue exclusion assay. Upon confirming cell death, the lysates were examined using the Pierce BCA protein assay kit (Thermo Fisher Scientific, Waltham, MA, USA) to determine antigen concentration. The lysates were then diluted to a stock concentration of 100 µg/mL in PBS and stored at −80 °C for subsequent re-stimulation studies. For T-cell proliferation assays, the prepared antigen was further diluted to create a series of concentrations, with the highest concentration at 5 µg/mL. For the evaluation of anti-tumor T cell responses, 5 µg/mL of the irradiated tumor antigen was used.

### 2.5. T Cell Antigen Stimulation

Single-cell suspensions of lymph node cells from tumor-bearing or non-tumor-bearing immunocompetent mice were generated by mashing lymph nodes through a 70 µm pore size nylon mesh in complete media. Cells were plated in 96-well plates in complete media at 1 × 10^5^ cells per well in a total volume of 200 µL. Cells were subsequently restimulated with LY2 or MOC2 tumor antigens for a period of 48 h, after which T cell proliferation and cytokine production were measured in culture supernatants.

### 2.6. T Cell Proliferation

T cell proliferation was determined by the reduction in resazurin using the Alamar Blue assay (Thermo Fisher Scientific). Briefly, lymph node cells from naïve or tumor-bearing mice were stimulated with LY2 and MOC2 tumor antigens prepared as described above, or with plate-bound αCD3 antibodies (1 µg/mL) and soluble αCD28 (1 µg/mL) antibodies in 96-well plates. After a 3-day incubation, an Alamar Blue assay was used to determine cellular proliferation.

### 2.7. Enzyme-Linked Immunosorbent Assay (ELISA)

The production of IL-4, IL-6, IL-10, IL-17, and IFN-γ was determined by ELISA as described previously [22], using the manufacturer’s protocol (Biolegend, San Diego, CA, USA). Capture and detection antibodies were purchased from Biolegend (San Diego, CA, USA).

### 2.8. Real-Time Quantitative PCR

Following exposure of LY2, MOC2, CAL27, and SCC83 cells to varying radiation doses, the cells were lysed using TRIzol. Subsequently, RNA extraction was performed using a Direct-zol RNA Miniprep kit (Zymo Research, Irvine, CA, USA). cDNA was synthesized using a High-Capacity cDNA Reverse Transcription Kit (Applied Biosystems, Foster City, CA, USA). Primer sequences were generated using the IDT Real-Time qPCR Tool (Integrated DNA Technologies, Coralville, IA, USA). Real-time PCR of cDNA samples was performed using the PowerUp SYBR Green Master Mix (Thermo Fisher Scientific, Foster City, CA, USA) with Actb and Gapdh as reference genes. Amplified gene transcripts include Ccl2, Ccl4, Ccl5, Cxcl9, Cxcl10, Cxcl11, and Cxcl12.

### 2.9. Statistical Analysis

Statistical analysis was performed using GraphPad Prism v8.0.2 (GraphPad Software, San Diego CA, USA). Analysis of variance or two-tailed Student’s T tests were used to determine statistically significant differences between groups using a pre-determined *p* value threshold of 0.05.

## 3. Results

### 3.1. Effect of Ionizing Radiation on HNSCC Cellular Proliferation

**Figure 1:**
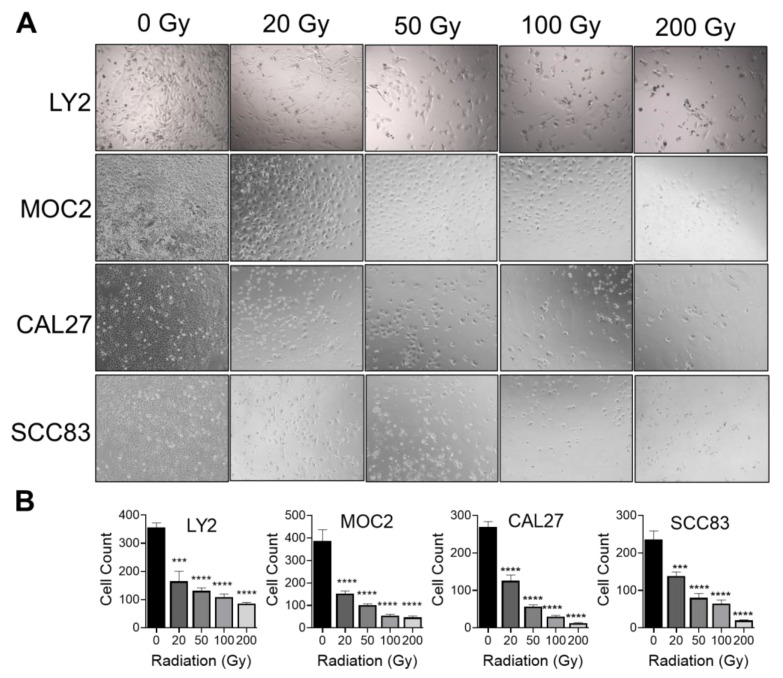
Effect of ionizing radiation on HNSCC cellular proliferation: (**A**,**B**) Dose-dependent decrease in viable mouse LY2, MOC2, and human CAL27 and SCC83 HNSCC cells with increasing doses of radiation from 0, 20, 50, 100, or 200 Gy. Data are presented as mean ± SE *** *p*-value < 0.001; **** *p*-value < 0.0001.

We first determined the impact of IR on the viability of human HNSCC cell lines (CAL27 and SCC83) and aggressive metastatic murine HNSCC cell lines LY2 and MOC2. Cells were allowed to grow to confluence prior to exposure to increasing doses of radiation between 0 and 200 Gy (Figure 1A). We observed a dose-dependent effect of IR on the growth of all HNSCC cells. Cells exposed to as few as 20 Gy saw a statistically significant reduction in cell number compared to cells not exposed to radiation, with the greatest reduction seen in cells exposed to 200 Gy (Figure 1B). IR doses as low as 20 Gy had significant impacts on the viability of all HNSCC cell lines.

### 3.2. Effect of Ionizing Radiation on Expression of T Cell Chemotactic Cytokines in HNSCC Cells

**Figure 2:**
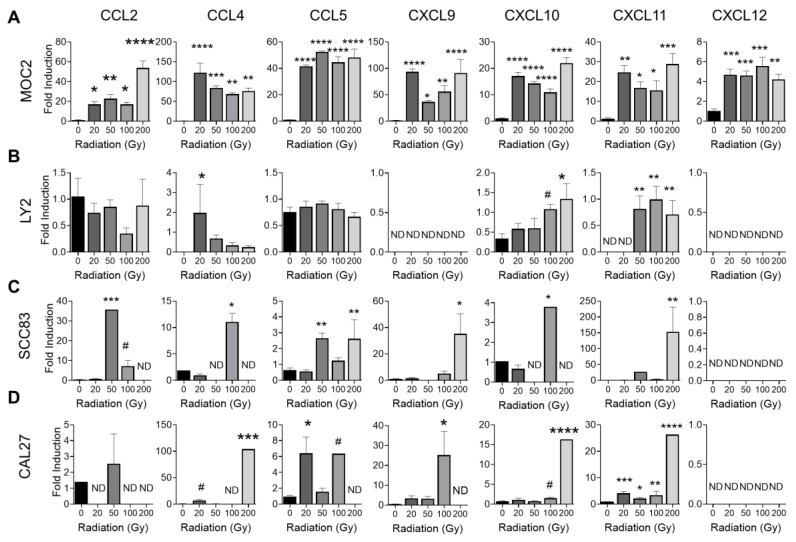
Effect of ionizing radiation on expression of T cell chemotactic cytokines in HNSCC cells. (**A**–**D**) Gene expression analysis of chemokines CCL2, CCL4, CCL5, CXCL9, CXCL10, CXCL11, and CXCL12 in control or irradiated (**A**) MOC2, (**B**) LY2, (**C**) SCC83, and (**D**) CAL27 HNSCC cells. HNSCC cells were exposed to various doses of radiation from 0, 20, 50, 100, or 200 Gy. Data are presented as mean ± SE # *p*-value < 0.1 * *p*-value < 0.05; ** *p*-value < 0.01; *** *p*-value < 0.001; **** *p*-value < 0.0001, when compared to control (non-irradiated) cells using ANOVA. ND—Not detected.

We next determined the effect of ionizing radiation on the expression of chemotactic cytokines known to promote the infiltration of T cell subsets to the solid tumor microenvironment. CXCL9, CXCL10, and CXCL11 are all ligands for the receptor CXCR3 that are expressed by CD8^+^ T cells and NK cells. Recognition of these ligands coordinates the recruitment of antitumoral CD8^+^ T cells and NK cells to the tumor site, where they may exert their anti-tumoral cytotoxic effector functions [23,24,25,26]. All doses of ionizing radiation (20 Gy to 200 Gy) significantly increased the expression of CXCL9, CXCL10, and CXCL11 in MOC2 cells (Figure 2A). In LY2 cells, high doses of ionizing radiation increased the expression of CXCL10 and CXCL11, while CXCL9 was not detected (Figure 2B). High doses of ionizing radiation induced the expression of CXCL9, CXCL10, and CXCL11 in human (SCC83 and CAL27) HNSCC cells (Figure 2C,D). These results suggest that ionizing radiation promotes antitumoral immune responses by inducing cytotoxic CD8^+^ T cell and NK cell homing to the HNSCC tumor microenvironment. Similarly, CCL5, which promotes CCR5-mediated CD8^+^ T cell infiltration to the primary tumor site [27], was upregulated in MOC2, SCC83, and CAL27, but not LY2 HNSCC cells (Figure 2A–D). CXCL12, which is involved in CXCR4-mediated recruitment of CD8^+^ T cell recruitment to the tumor microenvironment [24], was upregulated by ionizing radiation in MOC2 cells but not in LY2, SCC83, and CAL27 HNSCC cells (Figure 2A–D). Taken together, although various HNSCC cells differ in the chemokine receptor expression profile, T cell chemotactic chemokines are upregulated by ionizing radiation in both mouse and human HNSCC cells.

### 3.3. Effect of Various HNSCC Tumor Antigen Preparations on Anti-Tumor T Cell Responses

To determine the potential impact of HNSCC tumor cell irradiation on tumoral immune responses, we evaluated the effects of tumor antigens generated by IR, UV irradiation, or repeated freeze–thaw (F/T) cycles on the proliferation of tumor-antigen-specific T cells. T cells were isolated from draining lymph nodes of HNSCC tumor-bearing mice. We compared radiation effects with T cell activation via mechanical methods of tumor antigen preparation, such as repeated F/T of tumor cells, as well as polyclonal T cell activation [28,29]. Tumor antigens generated by IR were exposed to 200 Gy by X-ray ionizing radiation. Draining lymph node cells isolated from BALB/c LY2 or C57BL/6 MOC2 HNSCC tumor-bearing or non-tumor-bearing mice were exposed to either CD3/CD28 stimulating antibodies or HNSCC cell antigens prepared by one of the previously described methods. We further tested serial concentrations of HNSCC protein antigens generated by the various methods, which induced a proliferative T cell immune response, as determined by the Alamar Blue assay.

**Figure 3:**
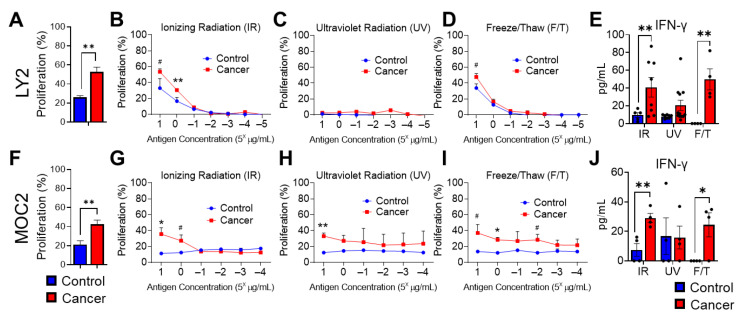
Effect of various HNSCC tumor antigen preparations on anti-tumor T cell responses. Lymph nodes from (**A**–**E**) LY2 and (**F**–**J**) MOC2 tumor (cancer) or non-tumor (control) bearing mice exposed to (**A**,**F**) CD3/CD28 stimulating antibodies or various concentrations of antigens generated by (**B**,**G**) ionizing radiation (IR), (**C**,**H**) ultraviolet radiation (UV), (**D**,**I**) freeze/thaw cycle (F/T). (**E**,**J**) IFN-γ levels in lymph node cells from tumor (cancer) or non-tumor (control) bearing mice after restimulation with IR, UV, and F/T antigen preparations as assessed by ELISA. Black dots represent data points from individual mice. Data are presented as mean ± SE # *p*-value < 0.1; * *p*-value < 0.05; ** *p*-value < 0.01.

In draining cervical lymph nodes, where T cell tumor immune responses are generated during HNSCC, conventional CD3/CD28 restimulation produced significantly increased T cell proliferation in tumor-bearing mice compared to non-tumor-bearing control mice (Figure 3A,F). IR-radiated tumor cell antigens also induced significant, dose-dependent antigen-specific cell proliferation in LY2 and MOC2 tumor-bearing mice compared to non-tumor-bearing control mice (Figure 3B,G). HNSCC tumor antigens generated by UV exposure produced negligible cellular proliferation in LY2 tumor-bearing mice, likely due to UV-induced disruptions of a higher-order protein structure (Figure 3C) [30]. Similarly, in MOC2 tumor-bearing mice, UV-generated tumor antigens did not sufficiently demonstrate dose-dependent antigen-specific T cell proliferation (Figure 3H). Tumor antigens generated by repeated F/T cycles induced significant cellular proliferation at higher protein concentrations but were less effective at discriminating tumor-specific versus non-tumor-specific cellular proliferation in LY2 tumor-bearing mice (Figure 3D). However, antigen-specific T cell proliferation was observed at various concentrations of F/T-generated MOC2 antigen preparation (Figure 3I). Together, our results demonstrate the consistent efficacy of IR-generated tumor antigens in the induction of tumor-specific T cell proliferation in draining lymph nodes in both LY2 and MOC2 orthotopic models of HNSCC.

Next, we determined the effect of these tumor antigen preparations on anti-tumor cytokine responses. Specifically, we examined interferon gamma production by lymph node cells restimulated with tumor antigens prepared by IR, UV, or F/T methods in tumor and non-tumor-bearing mice. We observed that, similar to cellular proliferation, tumor antigens prepared with IR and F/T, but not UV, produced the greatest amounts of IFN-γ in LY2 and MOC2 tumor-bearing mice, compared to non-tumor-bearing mice (Figure 3E,J).

### 3.4. Analysis of Antitumoral T Cell Immune Responses in Tumor Bearing Mice after Stimulation with Irradiated HNSCC Tumor Antigen Ex Vivo

**Figure 4:**
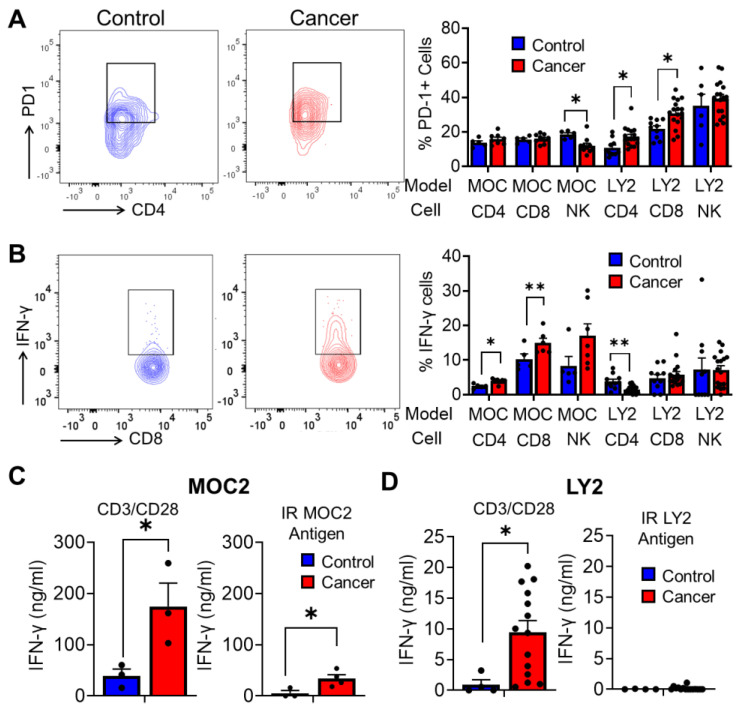
Analysis of antitumoral T cell immune responses in tumor-bearing mice after stimulation with irradiated HNSCC tumor antigen ex vivo. (**A**) Flow cytometry plots for PD1^+^ T cells with population frequencies for CD4^+^ and CD8^+^ T cells and NK cells in lymph node samples from tumor- and non-tumor-bearing mice. (**B**) Flow cytometry plots for IFN-γ^+^ T cells with population frequencies for CD4^+^ and CD8^+^ T cells and NK cells in lymph node samples from tumor- and non-tumor-bearing mice. (**C**) IFN-γ concentration of lymph node cells stimulated with CD3/CD28, or irradiated HNSCC tumor antigen (IR) in tumor (cancer) and non-tumor (control) bearing mice in (**C**) MOC2 and (**D**) LY2 HNSCC tumor models, as determined by ELISA. Black dots represent data points from individual mice. Data are presented as mean ± SE * *p*-value < 0.05; ** *p*-value < 0.01.

We chose two models of HNSCC in order to comprehensively examine the effect of ex vivo restimulation by IR-generated tumor antigens on antitumor immune responses. Our first model of HNSCC, LY2 HNSCC cells injected into STAT1-deficient BALB/c mice, is characterized by high levels of PD-1 expression and dampened IFN-γ production in lymph node CD4^+^ and CD8^+^ T cells [20] (Figure 4A,B). The second HNSCC model, MOC2 cells injected into wild-type C57BL/6 mice, is characterized by low levels of PD-1 expression and increased IFN-γ production (Figure 4A,B). We characterized the anti-tumor cytokine response of tumor-bearing and non-tumor-bearing mice after ex vivo restimulation with CD3/CD28 antibodies or IR HNSCC tumor antigen preparations in these models. In the MOC2 HNSCC model, IFN-γ production was significantly enhanced in lymph node cells of tumor-bearing mice stimulated with both CD3/CD28 antibodies and IR HNSCC antigens (Figure 4C). In the LY2 HNSCC model, we observed negligible but comparable levels of IFN-γ production in lymph node cells activated by IR HNSCC tumor antigen between tumor-bearing and non-tumor-bearing mice as determined by ELISA, corroborating our flow cytometric findings (Figure 4C). In contrast, lymph node cells activated by CD3/CD28 antibodies showed elevated amounts of IFN-γ tumor-bearing mice, compared to non-tumor-bearing mice (Figure 4D). Together, these results suggest that, compared to anti-CD3/CD28 restimulation, restimulation by IR HNSCC tumor cells more accurately represents T cell anti-tumor immune responses during experimental HNSCC carcinogenesis.

### 3.5. Analysis of T Cell Immune Responses Associated with HNSCC Progression after Ex Vivo Stimulation with Irradiated HNSCC Tumor Antigen

**Figure 5:**
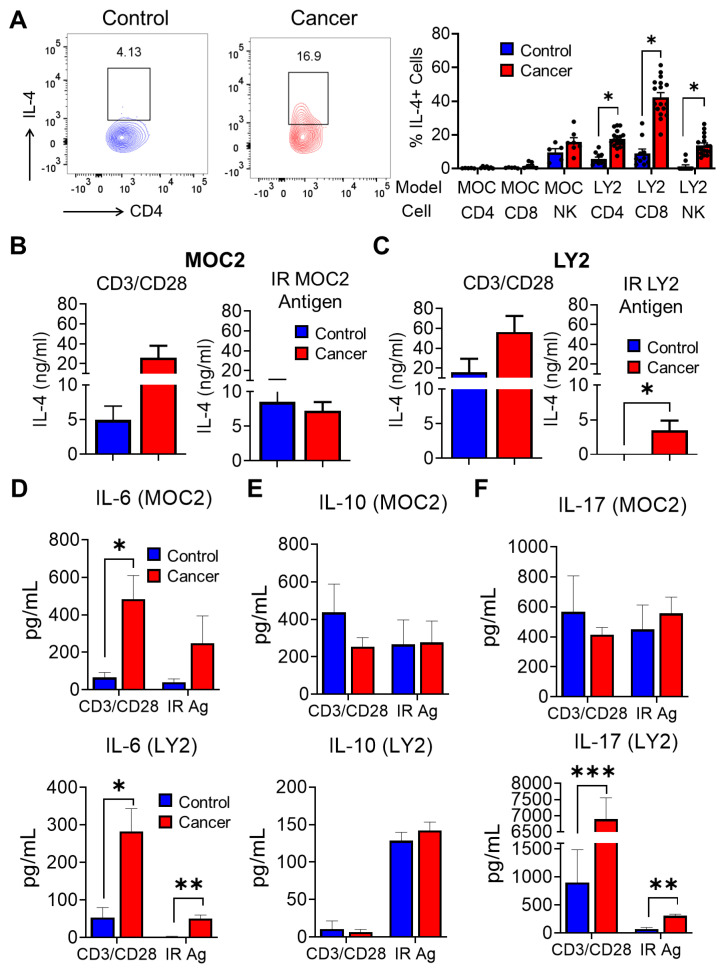
Analysis of T cell immune responses associated with HNSCC progression after ex vivo stimulation with irradiated HNSCC tumor antigen (**A**) Representative flow cytometry plots and population frequencies for IL-4^+^ CD4^+^ T cells and IL4^+^ CD8^+^ T cells in non-tumor bearing (control) and tumor bearing (cancer) lymph node cells. Black dots represent data points from individual mice. (**B**–**F**) Concentrations of (**B**,**C**) IL-4, (**D**) IL-6, (**E**) IL-10, and (**F**) IL-17 in lymph node cells stimulated with CD3/CD28, or irradiated HNSCC tumor antigen (IR Ag) in tumor (cancer) and non-tumor (control) bearing mice as determined by ELISA. Data are presented as mean ± SE * *p*-value < 0.05; ** *p*-value < 0.01; *** *p*-value < 0.001.

We next analyzed other cytokines associated with proinflammatory or anti-tumoral immune responses during HNSCC. Flow cytometric analysis of intracellular cytokine production revealed a greatly increased accumulation of CD4^+^ and CD8^+^ IL-4^+^ T cells in the draining lymph nodes of LY2-injected HNSCC tumor-bearing mice compared to non-tumor-bearing mice, but not in the MOC2 HNSCC model (Figure 5A). Similar to our flow cytometry results, lymph node cells from MOC2 tumor-bearing mice did not produce increased IL-4 compared to non-tumor-bearing mice after restimulation with IR MOC2 antigens (Figure 5B). However, lymph node cells from LY2 tumor-bearing mice stimulated with the IR LY2 tumor cell antigen ex vivo demonstrated increased secretion of IL-4 compared to non-tumor-bearing mice, as determined by cytokine ELISA (Figure 5C). This observation corresponds to the increased intracellular IL-4 observed via flow cytometric analyses. CD3/CD28 stimulated lymph node cells produced slightly higher but not significant levels of IL-4 in tumor-bearing compared to non-tumor-bearing mice in both LY2 and MOC2 HNSCC models (Figure 5B,C). We further investigated supernatants from CD3/CD28 or IR antigen-stimulated T cells for other cytokines relevant to HNSCC progression. The production of IL-6, capable of promoting tumor growth, was found to be increased in lymph node cells from tumor-bearing mice stimulated with CD3/CD28 antibodies compared to non-tumor-bearing mice in both LY2 and MOC2 HNSCC models (Figure 5D). IR LY2 antigen-stimulated lymph node cells from LY2 tumor-bearing mice demonstrated higher IL-6 production compared to non-tumor-bearing mice, but this effect was not observed in the MOC2 HNSCC model (Figure 5D). There were no differences in levels of the immunosuppressive cytokine IL-10 between lymph node cells of tumor-bearing or non-tumor-bearing mice in both LY2 and MOC2 HNSCC models. However, in the LY2 model, IL-10 was higher in lymph node cells stimulated with the IR tumor antigen compared to CD3/CD28 antibody stimulation, (Figure 5E). Draining lymph node cells showed an increased release of IL-17, known to promote HNSCC, in both CD3/CD28 and IR antigen stimulation conditions in the LY2 but not in the MOC2 HNSCC model (Figure 5F). In LY2-injected mice, the concentration of detectable IL-17 was considerably lower in IR tumor antigen-stimulated cells compared to cells stimulated with CD3/CD28 antibody (Figure 5F). Taken together, our data show that T cells exposed to the IR-derived tumor antigen demonstrate a cytokine profile that is more consistent with the tumoral immune response to experimental HNSCC in vivo, which makes this suitable as a tumor antigen for ex vivo immune assays.

## 4. Discussion

**Figure 6:**
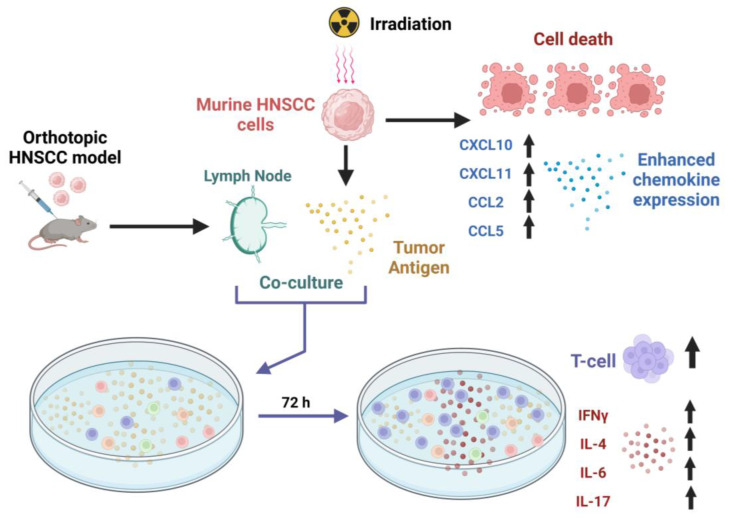
Schematic representation of effects of ionizing radiation on HNSCC cells and tumor-specific T cell immune responses (Created with BioRender.com). In addition to established inhibitory effects on tumor cell growth, ionizing radiation promotes the expression of T cell chemotactic cytokines by HNSCC cells and contributes to tumor-specific T cell proliferation and the induction of T cell cytokines.

Our results demonstrate the effect of radiation treatment on HNSCC cancer cells and show that IR in the range of 20–200 Gy produces a dose-dependent, cytotoxic effect on HNSCC cells. More significantly, we demonstrate that treating HNSCC with ionizing radiation produces immunogenic antigens that can induce anti-tumor-specific immune responses (Figure 6). These properties can be exploited in the rational design of combinatorial immunoradiotherapeutic strategies for HNSCC treatment. While these immunological effects are well established in other cancers, they are still yet to be fully characterized in HNSCC [31]. 

The promotion of cell death by IR has the potential to elicit innate and adaptive immune responses mediated by phagocytic cells, especially macrophages and dendritic cells, capable of clearing apoptotic cells [32] via specific surface receptors. Indeed, studies demonstrate that irradiated HNSCC cells are better phagocytized by M1 macrophages than non-irradiated HNSCC cells [33]. The subsequent antigen presentation of tumor antigens by macrophages to T cells initiates antitumor adaptive immunity. As shown by our study and others, IR potentially enhances T cell antitumor immune responses in the HNSCC tumor microenvironment [34]. A major mechanism of IR-mediated enhancement of T cell antitumor immune responses is the induction of anti-tumoral T cell chemotactic cytokines. Our results demonstrated that IR upregulated the expression of chemokines CXCL9, CXCL10, and CXCL11, which bind to CXCR3, mediating CD8^+^ T cell and NK cell recruitment to tumors [24,27].

Tumor antigens generated by IR were shown to elicit tumor-specific T cell proliferation compared to antigens generated by UV radiation. Although UV radiation can cause apoptotic death of cancer cells, it also induces permanent physiochemical damage to intracellular proteins, which may prevent the induction of adaptive immune responses that depend on the efficient presentation of polypeptide antigens [30]. Traditional freeze–thaw methods of antigen preparation appeared to work similarly to IR in promoting T cell proliferation and IFN-γ production ex vivo. This highlights the importance of maintaining the protein structure in the initiation of T cell immunity for laboratory studies. Future work will further characterize the nature of immune responses to freeze–thaw-generated tumor antigens. Our current study, which focuses on the immunogenic effects of IR HNSCC tumor antigens, demonstrates its efficacy in eliciting tumor-specific cellular proliferation and cytokine responses. Notably, the observed cytokine profile indicates that radiation-induced tumor cytotoxicity may help drive a Th1-skewed response, which is typically associated with anti-tumor activity [35,36]. T cells isolated from naive mice did not display these effects, demonstrating that this response was antigen-specific. Taken together, our results indicate that the direct killing of HNSCC cells with radiation therapy may contribute to a more robust anti-tumor immune response in patients, and this response can be optimized based on radiation dose. A deeper understanding of this antitumoral immune response will be critical in clinical applications of immune radiation combined therapies.

We employed two experimental models of HNSCC to better characterize the specific immunological responses associated with T cell stimulation using the IR HNSCC tumor antigen: (i) LY2 HNSCC cells injected into STAT1-deficient BALB/c mice [20,22] and (ii) MOC2 HNSCC cells injected into wild-type C57BL/6 mice [21,37,38]. STAT1 mediates IFNγ signaling and plays important anti-tumoral functions in the host immune response to HNSCC [39,40]. *Stat1*-deficient mice orthotopically injected with LY2 HNSCC cells are highly susceptible to tumor growth and metastasis and are associated with an exhausted T cell phenotype with enhanced PD1 expression [20]. T cells from tumor-bearing *Stat1*-deficient mice display attenuated IFNγ production and upregulation of immunosuppressive cytokines [20]. On the other hand, MOC2 cells injected into C57BL/6 mice promote increased IFNγ production by T cells in draining lymph nodes [21]. We, therefore, used both models to analyze cytokine responses after the stimulation of tumor-bearing and non-tumor-bearing lymph node cells with the IR-generated HNSCC tumor antigen or CD3/CD28 antibodies. Our data showed that CD3/CD28 stimulation of *Stat1*-deficient lymph node cells resulted in significantly elevated levels of IFNγ production. In contrast, stimulation with the IR HNSCC tumor antigen resulted in attenuated levels of IFNγ production. These results highlight the imprecise representation of the host immune response associated with artificial restimulation with CD3/CD28 antibodies. Irradiated HNSCC cells as tumor antigens provide a more accurate representation of the host tumor’s immune response status. This is demonstrated by our results showing comparable levels of IFNγ and IL-4 between ELISA detection of extracellular cytokines after ex vivo IR HNSCC stimulation and flow cytometric detection of intracellular cytokine production in PMA/ionomycin restimulated T cells.

The effects of IR on the immune response and cells of the tumor microenvironment further provide a strong rationale for combinatorial approaches with immunotherapy for HNSCC treatment [34,41,42,43,44]. Although radiotherapy primarily functions to induce cancer cell cytotoxicity via DNA damage and the generation of free radicals, it can also induce antitumor immune responses resulting in the indirect killing of tumor cells. IR has been shown to enhance the immunogenicity of HNSCC cells via the upregulation of the antigen processing and presentation machinery [18]. It promotes immunogenic cell death by stimulating the release of damage-associated molecular patterns, inducing the cGAS/STING pathway, which is important for the upregulation of type 1 interferons and crucial for dendritic cell activation and subsequent priming of T cells [45,46]. These properties of IR may facilitate a synergistic effect of combining IR with immunotherapy in HNSCC treatment [47]. Clinical evidence demonstrates that immunotherapy alone for HNSCC has met with limited success. As such, several clinical trials are being conducted to test the efficacy of combined radioimmunotherapy for HNSCC treatment [42,44,48]. In these contexts, functional evaluation of the tumor immune responses using irradiated tumor cells could potentially be of diagnostic or prognostic value. Furthermore, a deeper understanding of the immune response to irradiated tumor cells will facilitate more focused studies regarding the clinical application of immunoradiotherapy in HNSCC. Further research characterizing immunological responses to irradiated tumor antigens will be required to demonstrate the potential utility of this approach.

## 5. Conclusions

In conclusion, we demonstrate that HNSCC tumor antigens generated by ionizing radiation promote T cell responses predictive of the HNSCC tumor microenvironment. A deeper understanding of HNSCC patients’ immune responses to radiation therapy will facilitate the evaluation of patient outcomes and response to immunotherapy. IR-generated tumor antigens can potentially serve as a diagnostic indicator of antitumor immune responses in HNSCC.

## Data Availability

Data are contained within the article. The data presented in this study are available in this article.
